# Right Atrial Appendage Thrombus: “Can Be Found if You Look for It”∗

**DOI:** 10.1016/j.jaccas.2022.101702

**Published:** 2023-01-04

**Authors:** Miguel Ángel García-Fernández, Alberto Cresti

**Affiliations:** a Department Medicine, Faculty of Medicine, Complutense University, Madrid, Spain; b Department of Cardiology, Misericordia Hospital, Grosseto, Italy

**Keywords:** atrial fibrillation, echocardiography, thrombus

**Figure:**
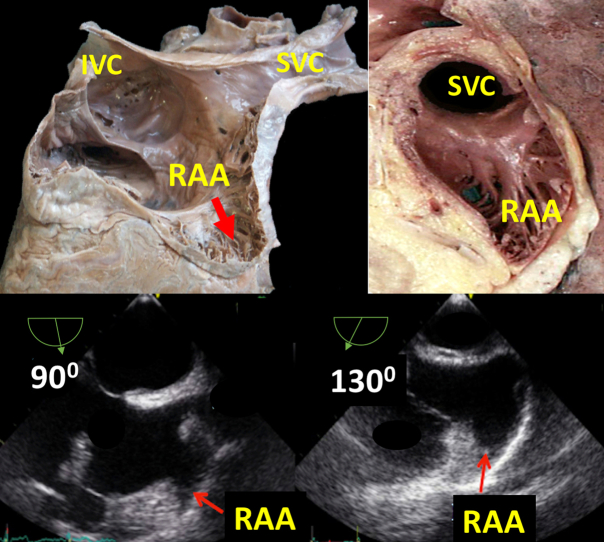

**Figure:**
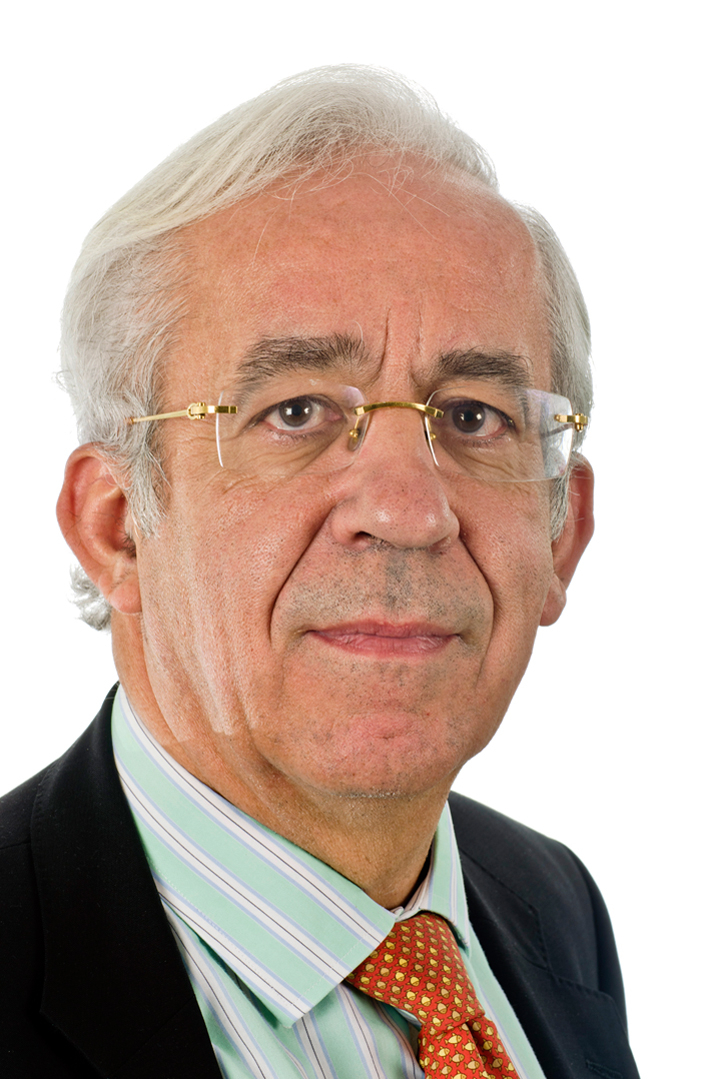

Atrial thrombi are a complication of atrial arrhythmias, fibrillation (AF), and flutter (AFL) and are mainly located in the left atrial appendage (LAA). The interest in atrial thrombosis localization in patients affected by AF is a topic that has regained new interest owing to the increasing use and feasibility of LAA occlusion techniques.

In this issue of *JACC: Case Reports*, Sakata et al^1^ describe a patient with persistent nonvalvular AF who could not tolerate anticoagulation therapy and underwent thoracoscopic left atrial appendectomy (TLAA) and thoracoscopic pulmonary vein isolation. Anticoagulation therapy was discontinued after both procedures, and 2 years later transthoracic echocardiography demonstrated a large right atrial appendage (RAA) mass. To confirm the diagnosis, the mass was surgically resected, and histopathologic examination revealed a thrombus of the RAA. The main message of this case report is to renew attention to the RAA, an unfairly forgotten part of the heart.

## LAA Thrombus in AF: TEE Detection

AF and AFL are the most common tachyarrhythmia in adults. They affect 2% to 3% of the population in North America and Europe, with 14% of the population over the age of 80 years old diagnosed.^2^^,^^3^ Apart from hemodynamic impairment, the significance of these arrhythmias is thromboembolic risk. It has been estimated that at least 1 of every 5 strokes may be caused by AF. Transesophageal echocardiography (TEE) is a sensitive tool for identification of thrombosis in the left atrium and LAA and TEE-guided cardioversion.

LAA is the most common site of atrial thrombosis, with a prevalence of 90% (in patients with LA clots), but this percentage refers to old studies, mostly in mixed populations of valvular and nonvalvular AF.^4^^,^^5^ The site of clot formation in AF has been reevaluated by our group in recent years and we have demonstrated,^6^ in a large population of consecutive patients (n = 1,420) affected by nonvalvular paroxysmal or persistent AF, that the prevalence of thrombosis not localized inside the LAA was 0.28%; therefore, the localization of thrombosis inside the LAA practically happens in 100% the cases. Left atrial cavity thrombosis is a very rare condition in nonvalvular AF.

## RAA: Normal Anatomy and Flow in TEE

**Figure 1:**
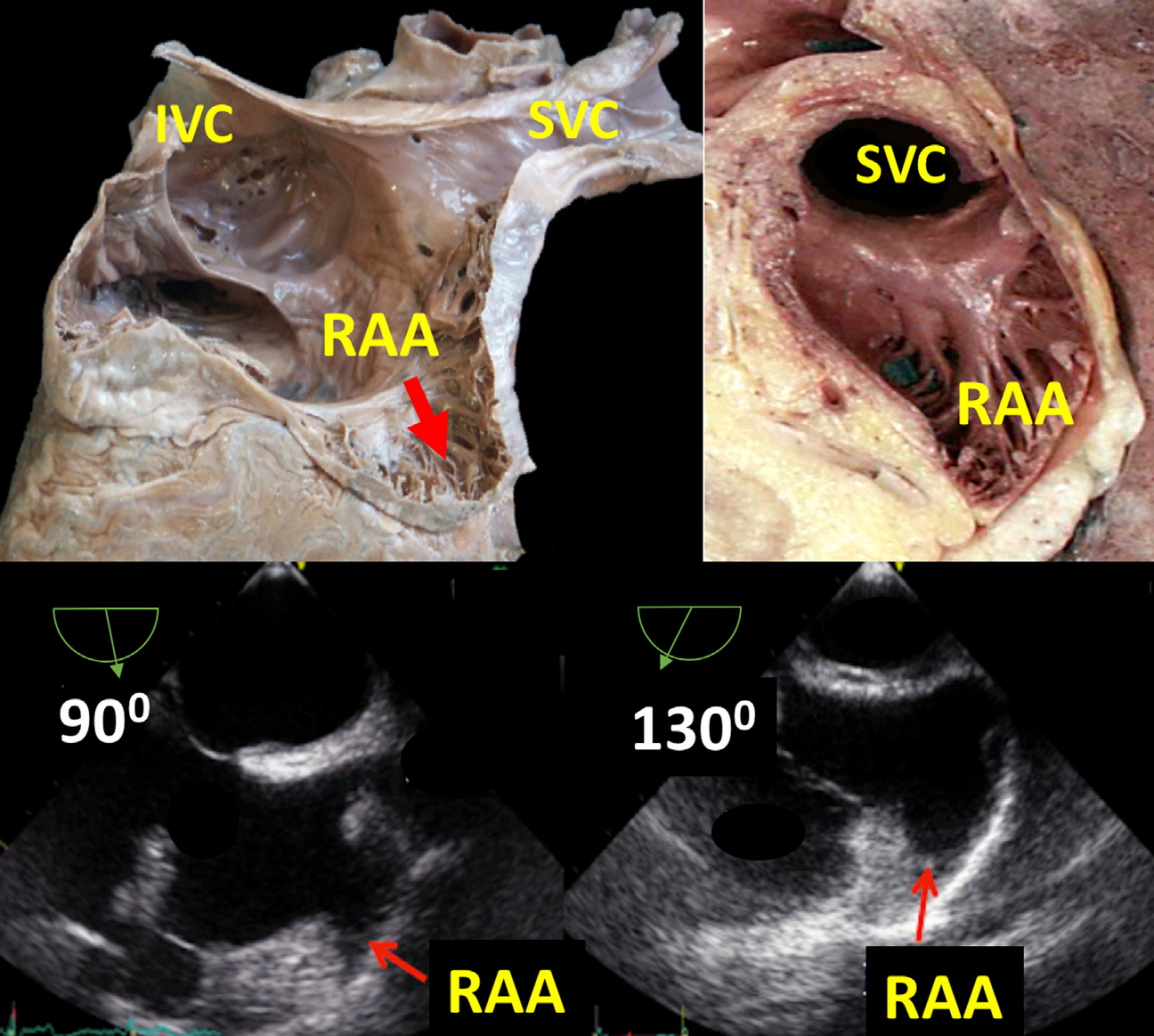
Anatomic and Transesophageal Echocardiographic View of Midesophageal Bicaval View at 90° and 130° of the Right Atrial Appendage IVC = inferior vena cava; RAA = right atrial appendage; SVC = superior vena cava.

RAA is a forgotten chamber, and its study, though quite simple, is rarely protocolized with TEE.^7^ With the midesophageal bicaval view at 90° and a rotation of the probe to 130°, the RAA can be entirely explored. RAA morphology was always unilobular and triangular shaped (Figure 1). Several different anatomic features characterize the RAA compared with the LAA. The LAA is an external appendage attached to the left atrium; in contrast, the RAA seems to be an integral prolongation of the right atrial cavity: it has a broad base, is shallow, and has a simple single triangle-shaped lobe with a smooth surface, whereas the LAA has a narrow neck, has a downward angle, is deep, and has a complex shape with multiple lobes and recesses. The inability to visualize the RAA in TEE has been reported to be as high as 16%,^2^ probably because operators are not used to exploring it.

The same LAA flow dynamics method, with pulsed-wave Doppler, can be applied to evaluate the filling and emptying flows in the RAA. We studied RAA Doppler in 38 patients in sinus rhythm and without valve disease undergoing TEE, and LAA and RAA peak outflow velocities were not statistically different; adequate RAA Doppler spectrum was not achievable in only 7% of TEE examinations.^7^

## RAA Thrombus in AF and AFL

TEE is the method of choice for the study and detection of thrombi in RAA.^7^ RAA thrombi are 12 times less frequent than LAA. In a series of 983 patients in which we performed TEE to guide AF cardioversion, the incidence of thrombosis was 9.3% in the LAA and 0.73% in the RAA. Maltagliati et al,^8^ in a study of 1,104 patients with persistent AF, found 6% LAA thrombi vs 0.4% RAA thrombi. In a recent series we studied 1,420 consecutive patients with paroxysmal or persistent atrial tachyarrhythmias,^6^ candidates for cardioversion, and there were 91 thrombi in 87 patients with a prevalence of 6.13%. In 3 cases, thrombi were in the RAA (3/87, 3.44%). Several different anatomic features of the RAA compared with the LAA, as previously described, may explain these differences in the appendage clots incidence. On the other hand, low flow velocity within the RAA, as assessed by pulsed-wave Doppler TEE, as well as the presence of spontaneous contrast in the RAA, are predictors of thrombus formation,^7^ similarly to LAA findings, in which low flow velocities and the presence of spontaneous contrast are predictors of thromboembolism and thrombus formation.^9^ Although there is no definitive information on the management of RAA thrombi, it is suggested that it should be the same as for LAA thrombi.^2^^,^^6^^,^^7^

RAA study does not add additional costs to TEE, it is not time consuming, and it is fast and well tolerated. We encourage LAA and RAA studies to avoid cardioversion and prescribe a full anticoagulation regimen when a thrombus is diagnosed.

## Atrial Thrombosis After TLAA

TLAA is, nowadays, a quite unusual intervention owing to the widespread feasibility of percutaneous LAA closure procedures. Therefore, there are no large-scale series on TLAA, and the frequency of atrial clots after this intervention cannot be estimated.

Undoubtedly, TLAA is potentially safe, and LAA removal is a relatively simple procedure that could be considered as an alternative option in patients with a contraindication to percutaneous closure of LAA.

For the same reasons, there are no clear guidelines on anticoagulant and antithrombotic regimens after TLAA nor on imaging follow-up. The patients described by Sakata et al^1^ did not undergo such therapies and did not undergo TEE evaluations after the procedure.

In the case of percutaneous LAA closure, a TEE “should be” performed, according to current guidelines, after 6 and 12-24 weeks and “may be” repeated after 1 year according to the patient’s characteristics and thrombophilic state,^10^ and it is mandatory whenever patients present a thromboembolic event.

In the case of surgical appendage excision, the potential thrombogenicity of the remaining appendage pouch is a matter of concern. In a nonrandomized study that compared the efficacy of several methods of LAA closure, TEE revealed a remnant LAA in 26% of patients, and one-half of them had suffered strokes in the time from the operation to when TEE was performed, despite none having clots in the remnant LAA.^11^ Thus, another important message arising from the case report by Sakata et al^1^ is the need for an expert consensus on appropriate follow-up imaging regimen after LAA excision, whether it was performed thoracoscopically or with open heart surgery.

## Funding Support and Author Disclosures

The authors have reported that they have no relationships relevant to the contents of this paper to disclose.
